# Rural–urban disparities in caesarean deliveries in sub-Saharan Africa: a multivariate non-linear decomposition modelling of Demographic and Health Survey data

**DOI:** 10.1186/s12884-022-04992-6

**Published:** 2022-09-17

**Authors:** Bright Opoku Ahinkorah, Richard Gyan Aboagye, Abdul-Aziz Seidu, Joshua Okyere, Aliu Mohammed, Vijay Kumar Chattu, Eugene Budu, Faustina Adoboi, Sanni Yaya

**Affiliations:** 1 School of Public Health, Faculty of Health, University of Technology Sydney, Sydney, Australia; 2 Department of Family and Community Health, Fred N. Binka School of Public Health, University of Health and Allied Sciences, Hohoe, Ghana; 3 College of Public Health, Medical and Veterinary Sciences, James Cook University, Townsville, Queensland Australia; 4 Centre For Gender and Advocacy, Takoradi Technical University, P.O.Box 256, Takoradi, Ghana; 5 Department of Population and Health, University of Cape Coast, Cape Coast, Ghana; 6 Department of Health, Physical Education and Recreation, University of Cape Coast, Cape Coast, Ghana; 7 Department of Community Medicine, Faculty of Medicine, Datta Meghe Institute of Medical Sciences, Wardha, Maharashtra 442107 India; 8 Department of Transdisciplinary Research, Saveetha Medical College and Hospitals, SIMATS, Saveetha University, TN Chennai, 600077 India; 9 Cape Coast Nursing and Midwifery Training College, Cape Coast, Ghana; 10 School of International Development and Global Studies, University of Ottawa, Ottawa, Canada; 11 The George Institute for Global Health, Imperial College London, London, UK

**Keywords:** Caesarean deliveries, Women, Decomposition analysis, Sub-Saharan Africa, Global health

## Abstract

**Introduction:**

Globally, the rate of caesarean deliveries increased from approximately 16.0 million in 2000 to 29.7 million in 2015. In this study, we decomposed the rural–urban disparities in caesarean deliveries in sub-Saharan Africa.

**Methods:**

Data for the study were extracted from the most recent Demographic and Health Surveys of twenty-eight countries in sub-Saharan Africa. We included 160,502 women who had delivered in health facilities within the five years preceding the survey. A multivariate non-linear decomposition model was employed to decompose the rural–urban disparities in caesarean deliveries. The results were presented using coefficients and percentages.

**Results:**

The pooled prevalence of caesarean deliveries in the 28 countries considered in the study was 6.04% (95% CI = 5.21–6.88). Caesarean deliveries' prevalence was highest in Namibia (16.05%; 95% CI = 14.06–18.04) and lowest in Chad (1.32%; 95% CI = 0.91–1.73). For rural-urban disparities in caesarean delivery, the pooled prevalence of caesarean delivery was higher in urban areas (10.37%; 95% CI = 8.99–11.75) than rural areas (3.78%; 95% CI = 3.17-4.39) across the 28 countries. Approximately 81% of the rural–urban disparities in caesarean deliveries were attributable to the differences in child and maternal characteristics. Hence, if the child and maternal characteristics were levelled, more than half of the rural–urban inequality in caesarean deliveries would be reduced. Wealth index (39.2%), antenatal care attendance (13.4%), parity (12.8%), mother’s educational level (3.5%), and health insurance subscription (3.1%) explained approximately 72% of the rural–urban disparities in caesarean deliveries.

**Conclusion:**

This study shows significant rural–urban disparities in caesarean deliveries, with the disparities being attributable to the differences in child and maternal characteristics: wealth index, parity, antenatal care attendance, mother’s educational level, and health insurance subscription. Policymakers in the included countries could focus and work on improving the socioeconomic status of rural-dwelling women as well as encouraging antenatal care attendance, women's education, health insurance subscription, and family planning, particularly in rural areas.

## Introduction

Despite the huge global interventions to reduce maternal mortality, it remains a major public health problem in many low- and middle-income countries (LMICs) [1]. In 2017 for instance, an estimated 295,000 maternal deaths were recorded worldwide, notwithstanding the steady decline in global maternal mortality rates over the past few decades according to the World Health Organisation (WHO) [2]. Most of these maternal deaths are preventable and occur mainly in LMICs (94%), with sub-Saharan Africa (SSA) alone accounting for almost 65% of the total global maternal deaths [2]. Although access to adequate emergency obstetric healthcare services such as caesarean delivery is associated with reducing maternal mortality [2], access to caesarean delivery is limited in most countries in SSA, especially among rural dwellers and women with lower socioeconomic status [3, 4].

Caesarean delivery is a surgical procedure that involves delivering one or more babies from a woman’s uterus [5]. It is one of the most important interventions for saving the lives of mothers and their foetuses during difficult labour [6]. The procedure is usually recommended when a vaginal delivery is likely to endanger the lives of the mother or foetus, such as during prolonged labour, foetal asphyxia [7], abnormal foetal presentation, antepartum haemorrhage and eclampsia [8]. Globally, the rate of caesarean deliveries has increased significantly from approximately 16.0 million (12.1% of all births) in the year 2000 to 29.7 million (21.1% of all births) in 2015 [8]. The increase in caesarean deliveries has largely been attributed to the growing rate of childbirths occurring at healthcare facilities due to improved access and the increasing trend of maternal requests for caesarean deliveries [6, 9–11]. The increasing trend of maternal requests for caesarean deliveries has become a global concern due to its exposure of women to short and long-term risk for maternal health complications [11, 12].

Meanwhile, there are wide disparities in the use of caesarean deliveries between and within countries. For instance, a recent global survey revealed that the prevalence of caesarean delivery varies from 0.6% in South Sudan to 58.1% in the Dominican Republic [8]. In SSA, approximately 3.0% of all births in Western and Central Africa and 4.6% in Eastern and Southern Africa occur through caesarean delivery [8]. Thus, despite the enormous increase in caesarean deliveries worldwide [12, 13], most countries in SSA still have caesarean delivery rates of less than 10% of total births in the population [9], which is lower than the WHO’s recommended 10–15% required for a reduction in both maternal and perinatal mortality [13]. The WHO suggests that a caesarean delivery rate of less than 10% indicates inadequate access to medically required caesarean deliveries [13], which increases the risk for maternal mortality.

Aside from the limited access to caesarean deliveries in SSA, there are vast within-country disparities in the use of caesarean deliveries, largely due to socio-economic and demographic inequalities [1, 9] Factors such as maternal age, education, wealth, parity, number of antenatal care visits, religion, ethnicity, health insurance coverage, employment status, reproductive history, and place of residence contribute significantly to the use of caesarean deliveries in SSA [1, 13, 14] In Nigeria, for example, higher odds for caesarean delivery were observed among women with a higher number of antenatal care visits, higher educational attainment, multiple pregnancies, higher household wealth, and among Christians [14]. In Burundi, higher rates of caesarean deliveries were observed among wealthy women, those with higher educational levels, and those living in urban areas [10].

Meanwhile, one of the major predictors for within-country differences in caesarean deliveries in SSA is the rural–urban differences in population characteristics [4, 15, 16]. Generally, higher rates of caesarean deliveries are recorded in urban areas and lower rates in rural areas [5]. Recent studies have shown that the higher rate of caesarean delivery among urban dwellers is largely due to the higher socio-economic status of women living in urban areas compared to those in rural areas [5, 15–17]. This is because women in rural communities are mostly poor and thus the least likely to receive adequate healthcare, including access to caesarean deliveries [2].

Although previous studies have investigated rural–urban differences in the use of caesarean deliveries in SSA [3, 4, 10, 14], most of these studies were conducted at the individual country level. Thus, there are limited multi-country analyses of rural–urban disparities in the use of caesarean deliveries in SSA. Having a comprehensive multi-country level data on the rural–urban differences in the use of caesarean deliveries could help in designing and implementing strategies that can improve access to medically required caesarean deliveries and perhaps contribute to achieving the Sustainable Development Goal (SDG) target 3.1 (i.e., achieving global maternal mortality ratio target of less than 70 per 100,000 live births by 2030). In this study, we decomposed the rural–urban disparities in caesarean deliveries in SSA.

## Methods

### Data source and study design

Data for the study were extracted from the most recent Demographic and Health Surveys (DHS) of twenty-eight countries in SSA. We pooled the data from the women's recode files in each of the 28 countries. The DHS is a comparatively nationally representative survey conducted in over 85 low-and-middle-income countries worldwide [18]. DHS employed a descriptive cross-sectional design. Respondents for the survey were recruited using a two-stage cluster sampling method. Detailed sampling technique has been highlighted in the literature [19]. Standardized structured questionnaires were used to collect data from the respondents on health indicators, including place and mode of delivery. We included a total of 160,502 women who had delivered in a health facility within the five years preceding the survey (Table 1). Only women with complete cases on the variables of interest in this study were included in the analyses. The dataset used is freely available at https://dhsprogram.com/data/available-datasets.cfm. This manuscript was drafted with reference to the Strengthening the Reporting of Observational Studies in Epidemiology (STROBE) statement guidelines [20].

**Table 1:** Description of the study sample

Countries	Year of survey	Weighted N	Weighted %
1. Angola	2015–16	5463	3.40
2. Burkina Faso	2010	10,046	6.26
3. Benin	2018	7,721	4.81
4. Burundi	2016–17	7,847	4.89
5. DR Congo	2013–14	8,876	5.53
6. Congo	2011-12	4,205	2.62
7. Cote d'Ivoire	2011–12	3,982	2.48
8. Cameroon	2018	5,045	3.14
9. Ethiopia	2016	7,066	4.40
10. Gabon	2012	2,187	1.36
11. Ghana	2014	3,400	2.12
12. Gambia	2019–20	4,437	2.76
13. Guinea	2018	4,858	3.03
14. Kenya	2014	5,487	3.42
15. Comoros	2012	1,471	0.92
16. Liberia	2019–20	2,452	1.53
17. Lesotho	2014	1,975	1.23
18. Mali	2018	5,776	3.60
19. Malawi	2015–16	10,995	6.85
20. Nigeria	2018	19,850	12.37
21. Namibia	2013	1,303	0.81
22. Sierra Leone	2019	5,108	3.18
23. Senegal	2010–11	6,343	3.95
24. Chad	2014–15	3,028	1.89
25. Togo	2013–14	4,270	2.66
26. Uganda	2016	7,921	4.94
27. Zambia	2018	5,212	3.25
28. Zimbabwe	2015	4,178	2.60
**All countries**	**2010–2020**	**160,502**	**100.00**

### Variables

#### Outcome variable

Caesarean delivery was the outcome variable in this study. With this variable, the women were asked the question, “Was (NAME) delivered by caesarean, that is, did they cut your belly open to take the baby out?”. The response options were “yes” and “no”. In the analysis, the response categories were recoded as “0 = no” and “1 = yes”. Studies that used the DHS dataset employed similar coding [1, 21].

#### Equity stratifier

Place of residence was the equity stratifier by which the disparity in caesarean delivery was measured. Previous studies have shown that place of residence plays a key role in caesarean delivery [5, 22, 23].

#### Explanatory variables

The main explanatory variable was place of residence. The responses for this were “rural” and “urban”.

### Covariates

The covariates considered in this study were selected based on their association with caesarean delivery from literature [1, 24–26] and their availability in the DHS dataset. The variables consisted of sex of the child, size of child at birth, twin status, mother’s age, educational level, current working status, marital status, religion, antenatal care attendance, national health insurance subscription, parity, partner’s educational level, person who usually decides on respondent’s health care, person who usually decides on large household purchases, person who usually decides on visits to family or relatives, sex of household head, frequency of reading newspaper or magazine, frequency of watching television, frequency of listening to the radio, and wealth index. The categories of each of the variables are shown in Table 2.

### Statistical analyses

Data for the study were analysed using Stata version 16. First, forest plots were used to show the prevalence of caesarean deliveries across the 28 countries and by the place of residence. Next, the distribution of caesarean section delivery across all the covariates was examined using chi-square test. The results were further disaggregated by place of residence. Third, multivariable binary logistic regression analysis was carried out to explore the predictors of caesarean deliveries. In the final analysis, a multivariate non-linear decomposition analysis [27] was employed to decompose the rural–urban disparities in caesarean deliveries. A multivariate decomposition analysis is used commonly in social research to quantify the contributions to group differences in average predictions from multivariate models. The technique uses the output from regression models to partition the components of a group difference in a statistic, such as a mean or proportion, into a component attributable to compositional differences between groups (that is, differences in characteristics or endowments) and a component attributable to differences in the effects of characteristics [27]. This technique was used to assess the variations in caesarean deliveries between rural and urban women and identify how much each of the covariates contributes to the variation. We applied the sample weights to obtain unbiased estimates according to the DHS guidelines. Also, the Stata survey command ‘svy’ was used to adjust for the complex sampling structure of the data in the chi-square and regression analyses. The variance inflation factor (VIF) was used to check for the presence of multicollinearity and there was no evidence of multicollinearity (mean VIF = 2.02, maximum = 4.51, minimum = 1.01).

### Ethical consideration

In this study, ethical clearance was not sought due to the public availability of the DHS dataset. The datasets were obtained from the Monitoring and Evaluation to Assess and Use Results Demographic and Health Survey (MEASURE DHS) after registration and approval were given for its usage. All the ethical guidelines concerning the use of secondary datasets in the publication were strictly adhered to. Detailed information about the DHS data usage and ethical standards are available at http://goo.gl/ny8T6X.

## Results

### Prevalence of caesarean deliveries among women in sub-Saharan Africa

**Fig. 1:**
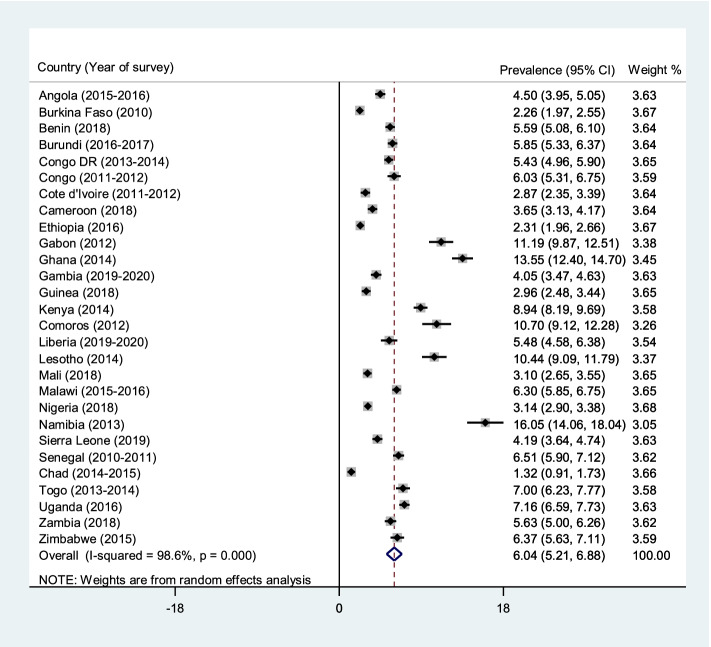
Forest plot showing the prevalence of caesarean deliveries in sub-Saharan Africa

**Fig. 2:**
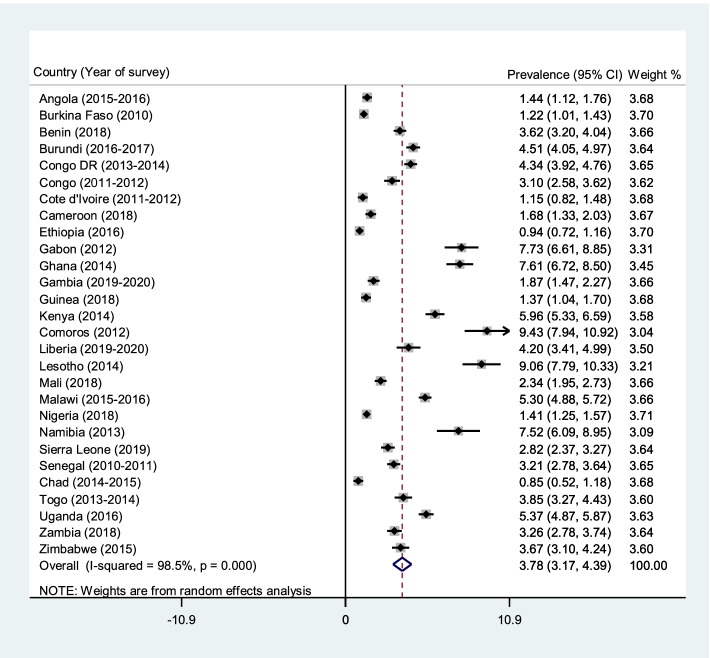
Forest plot showing the prevalence of caesarean deliveries in rural sub-Saharan Africa

**Fig. 3:**
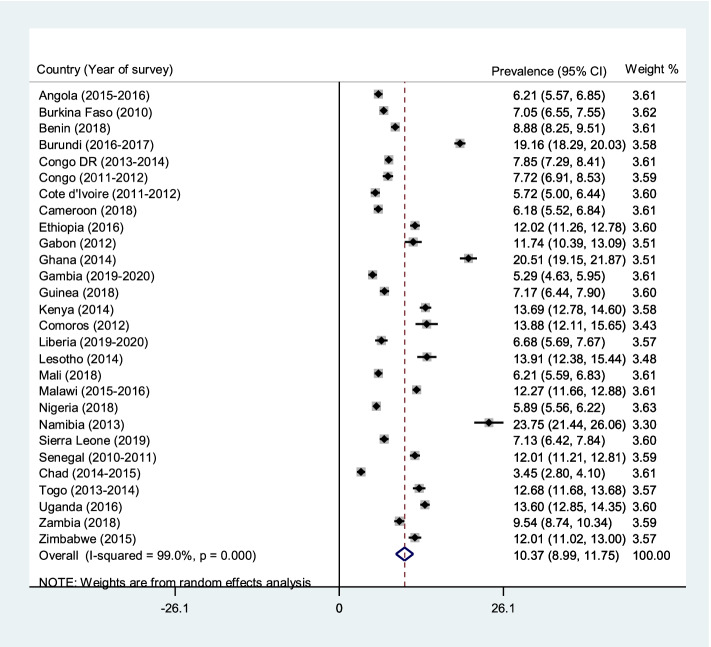
Forest plot showing the prevalence of caesarean deliveries in urban sub-Saharan Africa

The pooled prevalence of caesarean deliveries in the 28 countries considered in the study was 6.04% (95% CI = 5.21–6.88). Caesarean delivery was highest in Namibia (16.05%; 95% CI = 14.06–18.04) and lowest in Chad (1.32%; 95% CI = 0.91–1.73) (Fig. 1). For the rural-urban disparities in caesarean section, the pooled prevalence of caesarean delivery was higher in urban areas (10.37%; 95% CI = 8.99–11.75) than rural areas (3.78%; 95% CI = 3.17–4.39), and this observation was evident in all 28 countries (Figs. 2 and 3).

### Bivariable results of caesarean delivery among women in sub-Saharan Africa

Table 2 presents the bivariable results of caesarean delivery among women in SSA. Majority of the women had male children (50.8%), 48.2% had children who were average size at childbirth, and 97.9% had single birth children (Table 2). The modal age was 25–29 (26.5%), and the modal maternal level of education was no formal education (41.2%). Most of the women were working (65.9%), had four or more antenatal care visits (56.8%), were married (81.8%), Christians (57.5%), and were not covered by health insurance (93.5%). The modal parity was four or more births (47.6%), and modal partner educational level was no formal education (35.2%). Approximately 47.1% and 47.2% of the respondents’ partners decided alone on their healthcare and household purchases and 43.8% decided on visits to family or relatives with their partners (43.8%). Most of the respondents lived in male-headed households (86.2%), never watched television (62.1%), and never read newspaper/magazine (86.5%). The modal category for the frequency of listening to the radio was not at all (44.7%) and the modal wealth category was the poorest (21.7%). Statistically significant differences in caesarean delivery were observed across all the characteristics of the women (Table 2). Similar results were found across urban and rural areas. However, mother’s age showed no statistically significant difference in caesarean section in rural areas (Table 3).

**Table 2:** Bivariable analysis of caesarean deliveries among women in sub-Saharan Africa

Variables	Weighted N	Weighted %	Caesarean delivery		
			**No (%)**	**Yes (%)**	***p*-value**
**Sex of child**					< 0.001
Male	81,490	50.8	94.5	5.5	
Female	79,012	49.2	95.1	4.9	
**Mother’s self-reported size of child at birth**					< 0.001
Large	58,258	36.3	93.9	6.1	
Average	77,329	48.2	95.6	4.4	
Smaller	24,915	15.5	94.3	5.7	
**Twin status**					< 0.001
Single birth	157,123	97.9	95.0	5.0	
Multiple birth	3379	2.1	86.7	13.3	
**Mother’s age (years)**					< 0.001
15–19	9123	5.7	96.3	3.7	
20–24	33,680	21.0	95.7	4.3	
25–29	42,581	26.5	94.7	5.3	
30–34	34,240	21.3	94.3	5.7	
35–39	24,821	15.5	93.6	6.4	
40–44	12,066	7.5	94.8	5.2	
45–49	3991	2.5	95.2	4.8	
**Maternal educational level**					< 0.001
No education	66,121	41.2	97.5	2.5	
Primary	48,691	30.3	95.3	4.7	
Secondary	39,356	24.5	92.0	8.0	
Higher	6334	4.0	79.6	20.4	
**Current working status**					0.006
No	54,764	34.1	95.1	4.9	
Yes	105,738	65.9	94.6	5.4	
**Antenatal care attendance**					< 0.001
None	16,808	10.5	99.2	0.8	
1–3	52,552	32.7	96.5	3.5	
4 or more	91,142	56.8	93.0	7.0	
**Marital status**					< 0.001
Married	131,261	81.8	95.0	5.0	
Cohabiting	29,241	18.2	94.0	6.0	
**Religion**					< 0.001
No religion/others	4077	2.5	95.8	4.2	
Christianity	92,235	57.5	93.4	6.6	
Islamic	61,090	38.1	96.6	3.4	
African Traditional	3100	1.9	97.2	2.8	
**National health insurance subscription**					< 0.001
No	150,021	93.5	95.4	4.6	
Yes	10,481	6.5	86.2	13.8	
**Parity**					< 0.001
1 birth	27,226	17.0	91.7	8.3	
2 births	30,009	18.7	93.5	6.5	
3 births	26,928	16.8	94.4	5.6	
Four or more births	76,339	47.6	96.5	3.5	
**Partner's educational level**					< 0.001
No education	56,473	35.2	97.6	2.4	
Primary	41,801	26.0	95.6	4.4	
Secondary	49,354	30.8	93.1	6.9	
Higher	12,874	7.0	86.1	13.9	
**Person who usually decides on respondent’s health care**					< 0.001
Respondent alone	24,604	15.3	93.1	6.9	
Respondent and partner	59,270	36.9	93.8	6.2	
Partner alone	75,566	47.1	96.1	3.9	
Someone else or other	1062	0.7	95.9	4.1	
**Person who usually decides on large household purchases**					< 0.001
Respondent alone	19,161	11.9	93.4	6.6	
Respondent and partner	63,762	39.7	93.7	6.3	
Partner alone	75,799	47.2	96.0	4.0	
Someone else or other	1780	1.1	96.3	3.7	
**Person who usually decides on visits to family or relatives**					< 0.001
Respondent alone	30,451	19.0	94.2	5.8	
Respondent and partner	70,363	43.8	93.9	6.1	
Partner alone	58,428	36.4	96.1	3.9	
Someone else or other	1260	0.8	95.7	4.3	
**Sex of household head**					< 0.001
Male	138,375	86.2	95.0	5.0	
Female	22,127	13.8	93.4	6.6	
**Frequency of watching television**					< 0.001
Not at all	99,640	62.1	96.7	3.3	
Less than once a week	18,782	11.7	94.1	5.9	
At least once a week	42,080	26.2	90.5	9.5	
**Frequency of listening to radio**					< 0.001
Not at all	71,690	44.7	93.4	3.6	
Less than once a week	30,858	19.2	94.3	5.7	
At least once a week	57,954	36.1	93.0	7.0	
**Frequency of reading newspaper/magazine**					< 0.001
Not at all	138,833	86.5	95.8	4.2	
Less than once a week	12,235	7.6	90.0	10.0	
At least once a week	9434	5.9	86.4	13.6	
**Wealth index**					< 0.001
Poorest	34,832	21.7	98.1	1.9	
Poorer	34,395	21.4	97.3	2.7	
Middle	32,276	20.1	96.1	3.9	
Richer	30,944	19.3	93.8	6.2	
Richest	28,055	17.5	87.3	12.7	

^*^p-values are obtained from chi-square test

**Table 3:** Bivariable analysis of caesarean deliveries among women in sub-Saharan Africa segregated by place of residence

**Variables**	**Rural (*n* = 111,450)**				**Urban (*n* = 63,150)**			
	**Weighted**	**Caesarean section**			**Weighted**	**Caesarean section**		
	**N (%)**	**No (%)**	**Yes (%)**	***p*-value**	**N (%)**	**No (%)**	**Yes (%)**	***p*-value**
**Sex of child**				0.005				< 0.001
Male	56,724 (50.9)	96.6	3.4		24,782 (50.5)	90.2	9.8	
Female	54,726 (49.1)	96.9	3.1		24,270 (49.5)	91.5	8.5	
**Mother’s self-reported size of child at birth**				< 0.001				< 0.001
Large	39,275 (35.2)	96.1	3.9		18,854 (38.5)	89.9	10.1	
Average	54,056 (48.5)	97.2	2.8		23,312 (47.5)	92.1	7.9	
Smaller	18,119 (16.3)	96.6	3.4		6886 (14.0)	89.0	11.0	
**Twin status**				< 0.001				< 0.001
Single birth	109,197 (98.0)	96.8	3.2		47,936 (97.7)	91.1	8.9	
Multiple birth	2253 (2.0)	91.0	9.0		1116 (2.3)	78.9	21.1	
**Mother’s age (years)**				0.348				< 0.001
15–19	7087 (6.4)	96.8	3.2		2118 (4.3)	94.7	5.3	
20–24	24,306 (21.8)	96.5	3.5		9475 (19.3)	93.8	6.2	
25–29	28,364 (25.5)	96.7	3.3		14,085 (28.7)	91.1	8.9	
30–34	22,715 (20.4)	96.8	3.2		11,409 (23.2)	89.9	10.1	
35–39	16,965 (15.2)	96.7	3.3		7827 (16.0)	87.7	12.3	
40–44	8852 (7.9)	97.0	3.0		3266 (6.7)	89.6	10.4	
45–49	3161 (2.8)	97.3	2.7		872 (1.8)	88.7	11.3	
**Maternal educational level**				< 0.001				< 0.001
No education	55,710 (50.0)	98.1	1.9		11,484 (23.4)	94.8	5.2	
Primary	37,289 (33.5)	96.2	3.8		11,784 (24.0)	92.7	7.3	
Secondary	17,181 (15.4)	94.2	5.8		21,063 (43.0)	90.5	9.5	
Higher	1270 (1.1)	85.7	14.3		4721 (9.6)	78.1	21.9	
**Current working status**				0.049				< 0.001
No	36,844 (33.1)	96.9	3.1		17,790 (36.3)	91.7	8.3	
Yes	74,606 (66.9)	96.6	3.4		31,262 (63.7)	90.3	9.7	
**Antenatal care attendance**				< 0.001				< 0.001
None	15,135 (13.6)	99.4	0.6		2052 (4.2)	97.6	2.4	
1–3	41,502 (37.2)	97.3	2.7		11,599 (23.6)	93.9	6.1	
4 or more	54,813 (49.2)	95.5	4.5		35,401 (72.2)	89.4	10.6	
**Marital status**				< 0.001				0.013
Married	94,604 (87.9)	96.9	3.1		37,035 (75.5)	90.6	9.4	
Cohabiting	16,846 (15.1)	95.8	4.2		12,017 (24.5)	91.7	8.3	
**Religion**				< 0.001				< 0.001
No religion/others	3154 (2.8)	97.6	2.4		958 (1.9)	90.6	9.4	
Christianity	62,105 (55.7)	95.7	4.3		29,917 (61.0)	89.2	10.8	
Islamic	43,351 (38.9)	98.1	1.9		17,841 (36.4)	93.5	6.5	
African Traditional	2840 (2.6)	97.6	2.4		335 (0.7)	94.0	6.0	
**National health insurance subscription**				< 0.001				< 0.001
No	106,265 (95.3)	97.0	3.0		43,985 (89.7)	92.0	8.0	
Yes	5185 (4.7)	91.7	8.3		5067 (10.3)	81.1	18.9	
**Parity**				< 0.001				< 0.001
1 birth	17,146 (15.4)	94.6	5.4		9888 (20.2)	87.3	12.7	
2 births	18,743 (16.8)	96.0	4.0		11,037 (22.5)	89.8	10.2	
3 births	17,528 (15.7)	96.5	3.5		9272 (18.9)	91.0	9.0	
Four or more births	58,033 (52.1)	97.7	2.3		18,856 (38.4)	93.2	6.8	
**Partner educational level**				< 0.001				< 0.001
No education	47,759 (42.8)	98.2	1.8		9650 (19.7)	95.3	4.7	
Primary	33,889 (30.4)	96.5	3.5		8445 (17.2)	92.3	7.7	
Secondary	26,015 (23.3)	95.2	4.8		22,435 (45.7)	90.9	9.1	
Higher	3788 (3.4)	91.0	9.0		8522 (17.4)	84.2	15.8	
**Person who usually decides on respondent’s health care**				< 0.001				< 0.001
Respondent alone	15,587 (14.0)	95.6	4.4		8854 (18.1)	89.2	10.8	
Respondent and partner	40,273 (36.1)	96.1	3.9		18,900 (38.5)	89.4	10.6	
Partner alone	54,782 (49.2)	97.5	2.5		21,037 (42.9)	92.8	7.2	
Someone else or other	808 (0.7)	96.7	3.3		262 (0.5)	93.7	6.3	
**Person who usually decides on large household purchases**				< 0.001				< 0.001
Respondent alone	11,481 (10.3)	96.3	3.7		7480 (15.2)	89.4	10.6	
Respondent and partner	42,660 (38.3)	95.9	4.1		20,925 (42.7)	89.6	10.4	
Partner alone	56,132 (50.4)	97.5	2.5		20,051 (40.9)	92.5	7.5	
Someone else or other	1177 (1.1)	96.6	3.4		596 (1.2)	95.7	4.3	
**Person who usually decides on visits to family or relatives**				< 0.001				< 0.001
Respondent alone	19,536 (17.5)	96.8	3.2		10,739 (21.9)	90.0	10.0	
Respondent and partner	47,848 (42.9)	96.1	3.9		22,404 (45.7)	89.8	10.2	
Partner alone	43,137 (38.7)	97.5	2.5		15,572 (31.7)	92.8	7.2	
Someone else or other	929 (0.8)	95.7	4.3		337 (0.7)	95.7	4.3	
**Sex of household head**				< 0.001				< 0.001
Male	96,892 (86.9)	96.9	3.1		41,572 (84.8)	91.1	8.9	
Female	14,558 (13.1)	95.7	4.3		7480 (15.2)	89.4	10.6	
**Frequency of watching television**				< 0.001				< 0.001
Not at all	87,263 (78.3)	97.2	2.8		14,357 (29.3)	94.2	5.8	
Less than once a week	11,554 (10.4)	96.2	3.8		7066 (14.4)	91.1	8.9	
At least once a week	12,633 (11.3)	94.0	6.0		27,629 (56.3)	89.0	11.0	
**Frequency of listening radio**				< 0.001				< 0.001
Not at all	56,268 (50.5)	97.5	2.5		16,132 (32.9)	92.9	7.1	
Less than once a week	20,216 (18.1)	96.6	3.4		10,510 (21.4)	90.5	9.5	
At least once a week	34,966 (31.4)	95.6	4.4		22,410 (45.7)	89.5	10.5	
**Frequency of reading newspaper/magazine**				< 0.001				< 0.001
Not at all	102,913 (92.3)	97.0	3.0		36,633 (74.7)	92.6	7.4	
Less than once a week	5627 (5.1)	94.0	6.0		6294 (12.8)	86.9	13.1	
At least once a week	2910 (2.6)	91.0	9.0		6125 (12.5)	84.5	15.5	
**Wealth index**				< 0.001				< 0.001
Poorest	33,991 (30.5)	98.1	1.9		1915 (3.9)	97.1	2.9	
Poorer	31,730 (28.5)	97.4	2.6		3524 (7.2)	95.9	4.1	
Middle	24,982 (22.4)	96.4	3.6		7575 (15.5)	95.0	5.0	
Richer	15,568 (14.0)	94.9	5.1		14,728 (30.0)	92.8	7.2	
Richest	5179 (4.6)	90.3	9.7		21,310 (43.4)	86.6	13.4	

^*^p-values are obtained from chi-square test

### Rural–urban disparities in factors associated with caesarean delivery among women in sub-Saharan Africa

Overall, approximately 81% of the rural–urban disparities in caesarean section were attributable to the differences in child and maternal characteristics (Table 4). Hence, if the child and maternal characteristics were levelled, more than half of the rural–urban inequality in the caesarean section would be reduced. Among the child and maternal characteristics, wealth index (39.2%), antenatal care attendance (13.4%), parity (12.8%), educational level (3.5%), and health insurance coverage (3.1%) explained approximately 72% of the rural–urban disparities in caesarean section (Table 4). The likelihood of caesarean section increased with wealth index in both urban ([aOR = 2.83; 95% CI = 2.11–3.80] and rural areas [aOR = 2.58; 95% CI = 2.17–3.07]). However, the odds were slightly higher in urban areas. The likelihood of caesarean delivery decreased with increasing parity in both rural [aOR = 0.25; 95% CI = 0.21–0.29], and urban areas [aOR = 0.29; 95% CI = 0.25–0.34]. Compared to women who had no antenatal care, those who had four or more antenatal care visits were more likely to deliver through caesarean delivery, with higher odds in rural areas [aOR = 4.49; 95% CI = 3.42–5.89] compared to urban areas [aOR = 2.71; 95% CI = 1.80–4.11]. Women with a higher level of education were more likely to deliver through caesarean delivery than those with no formal education in both rural and urban areas. However, the odds were significant among women with higher education in rural areas only [aOR = 1.42; 95% CI = 1.15–1.76]. Women covered by health insurance were more likely to deliver through caesarean delivery than those who were not covered by health insurance in rural and urban areas. However, the odds were higher in rural areas [aOR = 1.65; 95% CI = 1.41–1.94] when compared to those in urban areas [aOR = 1.56; 95% CI = 1.39–1.75] (Table 5).

**Table 4:** Multivariate decomposition analysis of factors associated with caesarean section deliveries inequality between rural and urban residence

Variable	Difference due to Characteristics (E)		Difference due to Coefficients (C)	
	**Coefficient**	**Percent**	**Coefficient**	**Percent**
% Total explained disparity	0.04483***	81.12	0.01043***	18.88
**Sex of child**				
Male	-0.00000***	-0.00	0.00010	0.18
Female	-0.00000***	-0.00	-0.00010	-0.18
**Mother’s self-reported size of child at birth**				
Large	0.00010**	0.19	-0.00098**	-1.77
Average	0.00003***	0.06	-0.00023	-0.41
Smaller	-0.00019***	-0.35	0.00052**	0.94
**Twin status**				
Single birth	0.00004***	0.08	0.00141	2.55
Multiple birth	0.00004***	0.08	-0.00003	-0.05
**Mother’s age (years)**				
15–19	0.00085***	1.54	-0.00019	-0.34
20–24	0.00082***	1.49	-0.00165***	-2.98
25–29	-0.00059***	-1.07	-0.00114**	-2.06
30–34	0.00009	0.16	-0.00035	-0.64
35–39	0.00009***	0.16	0.00052*	0.94
40–44	-0.00048***	-0.86	0.00033	0.59
45–49	-0.00048***	-0.87	0.00028*	0.50
**Maternal educational level**				
No education	0.00186**	3.37	0.00028	0.51
Primary	0.00009	0.16	-0.00036	-0.65
Secondary	-0.00105**	-1.89	-0.00014	-0.26
Higher	0.00102***	1.85	0.00002	0.03
**Current working status**				
No	0.00001	0.03	-0.00037	-0.66
Yes	0.00001	0.03	0.00073	1.33
**Antenatal care attendance**				
None	0.00353***	6.39	0.00088*	1.59
1–3	-0.00131***	-2.37	-0.00097	-1.76
4 or more	0.00521***	9.42	-0.00187*	-3.39
**Marital status**				
Married	-0.00015*	-0.28	0.00207**	3.75
Cohabiting	-0.00015*	-0.28	-0.00040**	-0.73
**Religion**				
No religion/others	-0.00013*	-0.23	0.00012	0.22
Christianity	0.00020	0.37	-0.00032	-0.57
Islamic	0.00009*	0.16	0.00035	0.63
African Traditional	0.00015	0.26	-0.00012	-0.21
**National health insurance subscription**				
No	0.00086***	1.56	0.00049	0.88
Yes	0.00086***	1.56	-0.00002	-0.04
**Parity**				
1 birth	0.00186***	3.36	-0.00014	-0.26
2 births	0.00056***	1.02	0.00006	0.12
3 births	-0.00035***	-0.64	-0.00019	-0.34
Four or more births	0.00502***	9.08	0.00093	1.68
**Partner educational level**				
No education	0.00167**	3.02	0.00043	0.77
Primary	-0.00051*	-0.92	0.00036	0.66
Secondary	-0.00034	-0.62	-0.00041	-0.74
Higher	0.00065*	1.17	-0.00002	-0.04
**Person who usually decides on respondent’s health care**				
Respondent alone	-0.00014	-0.25	-0.00067	-1.20
Respondent and partner	-0.00004	-0.08	-0.00023	-0.41
Partner alone	0.00057	1.04	-0.00078	-1.41
Someone else or other	-0.00003	-0.05	0.00005	0.09
**Person who usually decides on large household purchases**				
Respondent alone	0.00027	0.48	0.00045	0.81
Respondent and partner	0.00028	0.50	0.00053	0.95
Partner alone	-0.00036	-0.64	0.00166	3.00
Someone else or other	-0.00000	-0.00	-0.00010	-0.19
**Person who usually decides on visits to family or relatives**				
Respondent alone	0.00034*	0.61	0.00155**	2.80
Respondent and partner	0.00034	0.62	0.00200	3.61
Partner alone	-0.00042	-0.75	0.00115	2.07
Someone else or other	0.00004	0.06	-0.00014*	-0.26
**Sex of household head**				
Male	0.00001	0.02	0.00098	1.77
Female	0.00001	0.02	-0.00015	-0.27
**Frequency of watching television**				
Not at all	0.00088	1.60	-0.00038	-0.69
Less than once a week	0.00011	0.19	0.00028*	0.52
At least once a week	-0.00016	-0.28	-0.00027*	-0.49
**Frequency of listening radio**				
Not at all	0.00036	0.65	0.00088	1.59
Less than once a week	0.00003	0.05	0.00003	0.05
At least once a week	0.00017	0.32	-0.00059*	-1.06
**Frequency of reading newspaper/magazine**				
Not at all	0.00062*	1.12	-0.00011	-0.21
Less than once a week	0.00005	0.08	0.00005	0.09
At least once a week	0.00028	0.50	-0.00002	-0.04
**Wealth index**				
Poorest	0.00630***	11.40	0.00039	0.71
Poorer	0.00234**	4.23	0.00036	0.65
Middle	0.00027	0.48	-0.00039	-0.71
Richer	0.00147***	2.66	-0.00013	-0.23
Richest	0.01127***	20.38	0.00002	0.03

^*^ *p* < 0.05, ^**^ *p* < 0.01, ^***^ *p* < 0.001

**Table 5:** Multivariable regression analysis of factors associated with caesarean deliveries among women in sub-Saharan Africa

Variables	Pooled AOR [95% CI]	Rural AOR [95% CI]	Urban AOR [95% CI]
**Sex of child**			
Male	1[1.00,1.00]	1[1.00,1.00]	1 [1.00,1.00]
Female	0.87^***^ [0.82,0.92]	0.89^**^ [0.82,0.97]	0.85^***^ [0.78,0.93]
**Twin status**			
Single birth	1[1.00,1.00]	1[1.00,1.00]	1 [1.00,1.00]
Multiple birth	3.74^***^ [3.23,4.34]	4.06^***^ [3.36,4.92]	3.52^***^ [2.83,4.39]
**Mother’s self-reported size of child at birth**			
Large	1[1.00,1.00]	1 [1.00,1.00]	1 [1.00,1.00]
Average	0.71^***^ [0.67,0.76]	0.68^***^ [0.62,0.75]	0.74^***^ [0.67,0.81]
Smaller	1.01 [0.92,1.10]	0.86^*^ [0.76,0.99]	1.15^*^ [1.01,1.31]
**Mother’s age (years)**			
15–19	1[1.00,1.00]	1 [1.00,1.00]	1 [1.00,1.00]
20–24	1.18^*^ [1.01,1.37]	1.25^*^ [1.04,1.51]	1.13 [0.87,1.47]
25–29	1.73^***^ [1.47,2.02]	1.67^***^ [1.37,2.04]	1.90^***^ [1.46,2.46]
30–34	2.35^***^ [1.98,2.79]	2.26^***^ [1.80,2.84]	2.61^***^ [1.99,3.42]
35–39	3.48^***^ [2.91,4.16]	2.84^***^ [2.24,3.61]	4.22^***^ [3.19,5.59]
40–44	3.43^***^ [2.81,4.19]	3.00^***^ [2.30,3.91]	4.03^***^ [2.95,5.50]
45–49	4.17^***^ [3.22,5.40]	3.11^***^ [2.21,4.36]	5.99^***^ [4.01,8.93]
**Maternal educational level**			
No education	1 [1.00,1.00]	1 [1.00,1.00]	1 [1.00,1.00]
Primary	1.18^***^ [1.07,1.31]	1.22^**^ [1.07,1.39]	1.04 [0.89,1.22]
Secondary	1.11 [0.99,1.25]	1.17 [1.00,1.37]	0.97 [0.82,1.15]
Higher	1.53^***^ [1.30,1.79]	1.31 [0.99,1.74]	1.41^**^ [1.15,1.74]
**Current working status**			
No	1 [1.00,1.00]	1 [1.00,1.00]	1 [1.00,1.00]
Yes	0.97 [0.91,1.04]	0.95 [0.86,1.04]	0.98 [0.89,1.09]
**Antenatal care attendance**			
None	1[1.00,1.00]	1 [1.00,1.00]	1 [1.00,1.00]
1–3	2.95^***^ [2.34,3.72]	3.33^***^ [2.54,4.37]	2.07^***^ [1.37,3.12]
4 or more	3.97^***^ [3.14,5.02]	4.49^***^ [3.42,5.89]	2.71^***^ [1.80,4.11]
**Marital status**			
Married	1 [1.00,1.00]	1 [1.00,1.00]	1 [1.00,1.00]
Cohabiting	1.03 [0.95,1.12]	1.15^*^ [1.02,1.30]	0.93 [0.83,1.05]
**Religion**			
No religion/others	1 [1.00,1.00]	1 [1.00,1.00]	1 [1.00,1.00]
Christianity	1.03 [0.84,1.25]	1.18 [0.91,1.53]	0.90 [0.68,1.20]
Islamic	0.79^*^ [0.65,0.98]	0.85 [0.65,1.13]	0.68^*^ [0.51,0.93]
African Traditional	1.01 [0.74,1.37]	1.20 [0.83,1.74]	0.88 [0.48,1.59]
**Parity**			
1 birth	1 [1.00,1.00]	1 [1.00,1.00]	1 [1.00,1.00]
2 births	0.62^***^ [0.56,0.68]	0.61^***^ [0.54,0.70]	0.61^***^ [0.54,0.69]
3 births	0.45^***^ [0.40,0.50]	0.45^***^ [0.39,0.52]	0.43^***^ [0.37,0.51]
Four or more births	0.27^***^ [0.24,0.30]	0.25^***^ [0.21,0.29]	0.29^***^ [0.25,0.34]
**National health insurance subscription**			
No	1 [1.00,1.00]	1 [1.00,1.00]	1 [1.00,1.00]
Yes	1.58^***^ [1.44,1.73]	1.65^***^ [1.41,1.94]	1.56^***^ [1.39,1.75]
**Partner educational level**			
No education	1 [1.00,1.00]	1 [1.00,1.00]	1 [1.00,1.00]
Primary	1.26^***^ [1.14,1.41]	1.20^**^ [1.05,1.37]	1.30^**^ [1.09,1.54]
Secondary	1.22^***^ [1.09,1.36]	1.21^**^ [1.05,1.39]	1.14 [0.97,1.33]
Higher	1.28^***^ [1.12,1.47]	1.42^**^ [1.15,1.76]	1.17 [0.98,1.41]
**Person who usually decides on respondent’s health care**			
Respondent alone	1 [1.00,1.00]	1 [1.00,1.00]	1 [1.00,1.00]
Respondent and partner	0.97 [0.88,1.07]	0.83^**^ [0.73,0.95]	1.08 [0.94,1.24]
Partner alone	0.89^*^ [0.80,0.98]	0.80^**^ [0.70,0.92]	0.95 [0.81,1.10]
Someone else or other	0.95 [0.62,1.44]	0.70 [0.42,1.17]	1.19 [0.58,2.43]
**Person who usually decides on large household purchases**			
Respondent alone	1 [1.00,1.00]	1 [1.00,1.00]	1 [1.00,1.00]
Respondent and partner	0.94 [0.84,1.04]	1.06 [0.91,1.25]	0.88 [0.76,1.01]
Partner alone	0.93 [0.83,1.04]	0.98 [0.84,1.14]	0.92 [0.79,1.07]
Someone else or other	0.70 [0.49,1.00]	0.96 [0.59,1.55]	0.52^*^ [0.30,0.91]
**Person who usually decides on visits to family or relatives**			
Respondent alone	1 [1.00,1.00]	1 [1.00,1.00]	1 [1.00,1.00]
Respondent and partner	1.02 [0.92,1.12]	1.12 [0.98,1.28]	0.96 [0.84,1.09]
Partner alone	1.01 [0.91,1.12]	1.08 [0.94,1.23]	0.97 [0.83,1.13]
Someone else or other	1.09 [0.74,1.61]	1.61^*^ [1.03,2.52]	0.58 [0.27,1.26]
**Sex of household head**			
Male	1 [1.00,1.00]	1 [1.00,1.00]	1 [1.00,1.00]
Female	1.11^*^ [1.02,1.20]	1.07 [0.96,1.20]	1.12 [0.99,1.27]
**Frequency of watching television**			
Not at all	1 [1.00,1.00]	1 [1.00,1.00]	1 [1.00,1.00]
Less than once a week	1.09 [0.98,1.21]	1.03 [0.89,1.18]	1.05 [0.90,1.24]
At least once a week	1.13^**^ [1.03,1.24]	1.14^*^ [1.00,1.30]	1.05 [0.93,1.20]
**Frequency of listening radio**			
Not at all	1 [1.00,1.00]	1 [1.00,1.00]	1 [1.00,1.00]
Less than once a week	1.07 [0.98,1.17]	1.06 [0.94,1.20]	1.07 [0.94,1.22]
At least once a week	1.09^*^ [1.01,1.18]	1.16^**^ [1.04,1.29]	1.05 [0.94,1.17]
**Frequency of reading newspaper/magazine**			
Not at all	1 [1.00,1.00]	1 [1.00,1.00]	1 [1.00,1.00]
Less than once a week	1.10 [0.99,1.21]	1.00 [0.85,1.17]	1.15^*^ [1.02,1.30]
At least once a week	1.21^**^ [1.08,1.35]	1.28^*^ [1.06,1.55]	1.23^**^ [1.07,1.41]
**Wealth index**			
Poorest	1 [1.00,1.00]	1 [1.00,1.00]	1 [1.00,1.00]
Poorer	1.23^***^ [1.10,1.38]	1.17^**^ [1.05,1.34]	1.33 [0.94,1.88]
Middle	1.55^***^ [1.39,1.74]	1.48^***^ [1.31,1.68]	1.53^**^ [1.12,2.08]
Richer	2.07^***^ [1.84,2.33]	1.85^***^ [1.61,2.13]	2.02^***^ [1.50,2.72]
Richest	2.97^***^ [2.62,3.36]	2.58^***^ [2.17,3.07]	2.83^***^ [2.11,3.80]

Exponentiated coefficients; 95% confidence intervals in brackets; ^*^ *p* < 0.05, ^**^ *p* < 0.01, ^***^ *p* < 0.001; *AOR* Adjusted Odds Ratio, *CI* Confidence Interval; 1[1.00,1.00] = Reference category

## Discussion

The current study sought to decompose the rural–urban differences in the use of caesarean deliveries in SSA. Generally, a prevalence of < 9% is considered low prevalence of caesarean deliveries [28]. We found an overall prevalence of 6.04%, which corroborates previous studies that have showed that countries in SSA have a low prevalence (6%) of caesarean deliveries [29, 30]. The observed low prevalence of caesarean deliveries in SSA reflects Miller et al.’s *“too little, too late”* [31], which links low prevalence of caesarean deliveries to lower rates of institutional deliveries and deficiencies in resources and evidence-based care. We found the highest prevalence in Namibia, whereas Chad reported the lowest prevalence of caesarean deliveries. It is important to note that Namibia’s prevalence (16.05%) is a little above the accepted interval (5–15%) by the WHO [1].

Our findings indicate rural–urban disparities in caesarean deliveries among women in the 28 countries included in the study, with urban areas reporting a higher prevalence of caesarean deliveries. The result supports previous studies that have reported that urban areas have a disproportionately higher prevalence of caesarean deliveries than rural areas [10, 32]. This could be due to the comparative advantage urban residences have over rural areas regarding access to obstetric care [33]. Nonetheless, we found that over two-thirds of the rural–urban disparities in caesarean deliveries were attributable to the differences in child and maternal characteristics, including wealth index, parity, antenatal care attendance, educational level, and health insurance coverage.

We found differences in the prevalence of caesarean deliveries attributable to the wealth index, which aligns with the findings of a related study in Ethiopia [32]. Rural areas are usually disadvantaged in access to obstetric care, with the nearest health facilities being miles away from such communities [33]. This means that poorer women in rural areas would find it difficult to afford transportation to access facility birthing, let alone utilize caesarean section. Although there is the user fee exemption policy in most SSA countries including Ghana and Nigeria, evidence suggests that there are substantial inequalities in access to caesarean deliveries with women in lower wealth index having significantly lower likelihood to have a by caesarean deliveries [34]. The observed result also corroborates the findings from our multivariable regression analyses that showed a significant association between wealth index and the odds of caesarean deliveries in both the urban and rural settings, with greater odds being reported in urban areas. This implies that enhancing the socio-economic status of women in rural areas to attend antenatal care attendance and seek facility birthing may help close the rural–urban gap in caesarean deliveries.

Consistent with the findings of a related study by Lisonkova et al. [35], rural–urban disparities in the prevalence of caesarean deliveries were attributable to the differences in parity of women. Additionally, our regression analyses also revealed that the odds of caesarean deliveries significantly declined with increasing parity in both rural and urban settings. However, the odds were much lower in rural areas as opposed to those in urban areas. Our result is supported by a related study from Ghana [36] that reported significantly lower odds of birth by caesarean deliveries among multiparous women in both rural and urban settings as compared to uniparous women. Evidence shows that once women start birthing, subsequent deliveries become less risky until they reach the point of grand-multipara (i.e., their fifth delivery) [37]. This phenomenon could possibly explain why the likelihood of caesarean deliveries reduced significantly with increasing parity in both rural and urban settings. Nevertheless, the relatively lower odds of delivering by caesarean deliveries in rural areas as opposed to those in the urban areas is an indication of “too little, too late” as opined by Miller et al. [31]. That is, a situation where women who are in need of caesarean deliveries are unable to access it or that they get access to caesarean deliveries late, probably because of the distance to healthcare facilities, and problem with paying for the cost.

Antenatal care emerged an important maternal characteristic that explained the differences in rural–urban disparities in caesarean deliveries. Greater odds of birth by caesarean deliveries were reported among women who had 4 or more antenatal care visits in both rural and urban residencies, with much higher likelihoods in rural settings than urban-dwelling women. The results are in agreement with a study from Nigeria [33] that reported two times greater odds of delivering by caesarean deliveries among women with 4 + antenatal care visits. This observation may be explained from the perspective that 4 + antenatal care visits offer the opportunity for healthcare providers to detect pregnancy complications and identify women who may need to deliver by caesarean deliveries [38]. Also, it serves as a conduit to create awareness of caesarean deliveries and facilitate women’s capacity to make an informed decision to undergo elective caesarean section [33, 39]. It is also possible that women with more than 4 antenatal care visits may also have a higher propensity to seek care and to adhere to healthcare providers' recommendations. Therefore, it implies that interventions aimed at improving caesarean deliveries utilisation would have to encourage women in rural areas to attend antenatal care to help close the rural–urban gap in caesarean deliveries.

We also found that educational attainment explains the rural–urban differences in caesarean deliveries deliveries. Generally, higher educational attainment is associated with better socio-economic status, higher knowledge about healthcare services and greater autonomy of healthcare decision-making [33]. Hence, similar dynamics play out in birthing by caesarean deliveries. Additionally, this finding confirms our result that women with higher level of education was more likely to deliver through caesarean deliveries than those with no formal education in rural and urban areas. However, we found that the odds were relatively higher in rural areas than those in urban areas. There is consensus in the literature that rural dwelling women are often disproportionately disadvantaged in terms of higher levels of education and health-seeking [40]. Therefore, improving the educational level of women in rural areas provides an avenue for women who require caesarean deliveryto have access to it.

The likelihood of delivering by caesarean deliveries was significantly higher among women covered by health insurance in rural and urban areas; however, the odds were higher among rural-dwelling women. Available evidence indicates that poor socio-economic status is a significant barrier to the utilisation of caesarean deliveries by women who need it; thus, demonstrating a scenario of *“too little, too late”* [31]. As such, health insurance coverage offsets this barrier by significantly limiting out-of-pocket payment, promoting greater appeal and odds of utilising caesarean deliveries [33]. Our study further revealed that rural women covered by health insurance have the same caesarean deliveries rates than urban women not covered by health insurance (8%). Moreover, urban women covered by health insurance present very high caesarean deliveries rates (19%). Thus, reflecting a situation of *“too much, too soon”*. That is, a situation where there are more caesarean deliveries than needed.

### Strength and limitations

The national representativeness of the DHS data ensures that our findings can be generalized to women in the 28 included countries. Nevertheless, our study has some limitations that should be considered when interpreting the findings. The DHS does not include women who got caesarean deliveries with stillbirth in the samples, and therefore, the prevalence of caesarean deliveries as reported in this study may not be the true reflection of the reality. Also, the DHS is based on the cross-sectional design and thus, we were able to also establish association but not causal inferences. Finally, the differences in surveys years for the various countries could affect comparisons of estimates across countries. Another limitation is that, we excluded all births that occurred outside the healthcare facilities. Therefore, any interpretation of our findings note that our analysis is facility-based rather than population based.

## Conclusion

We found significantly low prevalence of caesarean deliveries among the 28 SSA countries. Findings from this study suggest significant rural–urban disparities with respect to caesarean deliveries, with the disparities being attributable to the differences in maternal and child characteristics: wealth index, parity, antenatal care attendance, educational level, and health insurance coverage. Therefore, policymakers in the included countries could focus and work on improving the socioeconomic status of rural-dwelling women as well as encouraging antenatal care attendance, women's education, health insurance coverage, and family planning, particularly in rural areas.

## Data Availability

The data for this study can be accessed on https://dhsprogram.com/data/available-datasets.cfm.

## Abbreviations

ANC: Antenatal care
CS: Caesarean section
DHS: Demographic and Health Survey
LMICs: Low-income and Middle-income Countries
SDG: Sustainable Development Goal
SSA: Sub-Saharan Africa
STROBE: Strengthening the Reporting of Observational Studies in Epidemiology
